# 2025 EP Fellows Summit: A Year of Innovation and Clinical Excellence

**DOI:** 10.19102/icrm.2026.17044

**Published:** 2026-04-15

**Authors:** William Sauer, Wendy Tzou


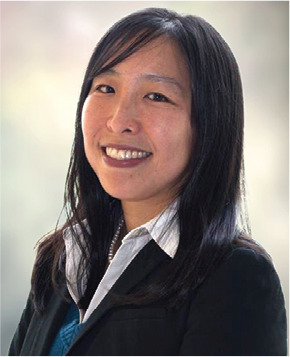



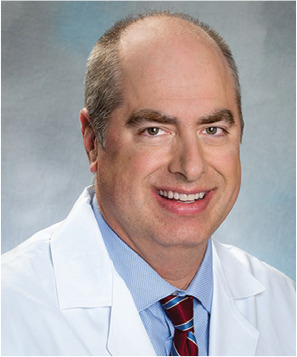


Dear readers,

As the Directors of the Electrophysiology Fellows Summit and the Arrhythmia Scholars Program, we are proud to introduce the published case reports of the three finalists who were selected to present their unique cases during the Case Competition at the 2025 EP Fellows Summit held November 7–9, 2025, in Reston, VA.

As in previous years, we received numerous outstanding case reports this past year to judge. After a thorough review of the exceptional case entries submitted by fellows and residents from around the globe, the program committee nominated three finalists to present their work and answer questions from the panel of judges. The winner was announced at the sessions’ conclusion following the panel deliberation and received a trophy and a cash prize.

Dr. Hasan Munshi^1^ from St. Joseph’s University Medical Center in Paterson, NJ, presented a compelling case of persistent right phrenic nerve palsy following pulsed field ablation in a patient with persistent atrial fibrillation. Their case report detailed the challenging management of a 61-year-old woman who developed this rare complication after pulmonary vein isolation and posterior wall isolation using a PulseSelect™ catheter (Medtronic, Minneapolis, MN, USA). The presentation highlighted the importance of vigilance for collateral injury even with tissue-selective ablation technologies and emphasized the need for structured follow-up to optimize patient outcomes.

Dr. Jonathan Gordon^2^ from Rush University Medical Center in Chicago, IL, described an innovative approach to treating incessant ventricular fibrillation in a patient with a left ventricular assist device (LVAD). His case demonstrated the successful use of Purkinje fiber catheter ablation targeting short-coupled premature ventricular complexes that triggered ventricular fibrillation. The presentation showcased how high-power, short-duration radiofrequency ablation can effectively debulk Purkinje fibers, offering a novel anatomical approach that may reduce procedural time in critically ill patients on mechanical support.

Finally, Dr. Sittinun Thangjui^3^ from West Virginia University presented the winning case on the feasibility of combined pulsed field and radiofrequency ventricular tachycardia ablation using the Sphere-9™ catheter (Medtronic) guided by EnSite™ mapping (Abbott, Chicago, IL, USA) in a patient with a durable LVAD. This groundbreaking case demonstrated the first successful use of this hybrid approach in an LVAD patient, overcoming the electromagnetic interference limitations of magnetic-based mapping systems. The combination of pulsed field ablation and radiofrequency energy offered a novel strategy for complex ventricular tachycardia substrate modification, particularly in regions adjacent to prosthetic structures where thermal injury risk is high.

Congratulations to Dr. Thangjui on his winning case and to Drs. Munshi and Gordon as the case competition finalists for their unique and innovative case presentations.

We look forward to the 2026 EP Fellows Summit scheduled for November 13–15, 2026, in Reston, VA. As a hybrid conference, attendees will have the choice of attending the Summit virtually or as a traditional in-person event near Washington, DC, with the chance to participate in hands-on training sessions and interpersonal engagement. For those who are unable to attend the Summit, virtual attendance and engagement will be made possible from the convenience of your computer or mobile device through the Summit’s innovative livestreaming broadcast platforms. Detailed information will be made available at www.epfellowssummit.com.

Sincerely,

William Sauer, md

Brigham and Women’s Hospital

Harvard Medical School

Boston, MA, USA

and

Wendy Tzou, md

University of Colorado Anschutz Medical Campus

Aurora, CO, USA
